# Impact of body weight and age on plantar pressure in typically developing children: Normative data and methodological considerations

**DOI:** 10.1177/18632521251335875

**Published:** 2025-05-31

**Authors:** Anika Behrendt, Tobias Siebert, Sonia D’Souza

**Affiliations:** 1 Motion and Exercise Science, University of Stuttgart, Stuttgart, Germany; 2 Stuttgart Center for Simulation Science (SC SimTech), University of Stuttgart, Stuttgart, Germany; 3 Gait Laboratory, Orthopaedic Clinic, Olga Hospital, Klinikum Stuttgart, Stuttgart, Germany

**Keywords:** Pedobarography, normative data, parameter normalisation, children, body weight, chronological age

## Abstract

**Purpose:**

Pedobarography is frequently employed for the identification and characterisation of foot pathologies in paediatrics. However, the lack of standardised normalisation methods presents a challenge for cross-age comparisons. This cross-sectional study provides normative plantar pressure data for typically developing children aged 4–17 years and compares normalisations and explanatory powers of parameters measuring peak and total load.

**Methods:**

Dynamic foot pressure of 101 typically developing children aged 4–17 years was measured at self-selected speed using the mid-gait protocol. They were divided into five age groups: 4–6, 7–8, 9–11, 12–14 and 15–17 years old. Force and pressure variables measuring peak and total load were normalised by body weight or scaled by maximum value and the foot region where the peak pressure occurred was identified.

**Results:**

The absolute values demonstrated an increase in load with advancing age. In contrast, when normalised to body weight, peak pressure and pressure time integrals decreased. The scaled peak pressure showed a load shift to the forefoot. The results indicate that the normalised parameters exhibit superior qualitative significance, suggesting a more dynamic gait pattern and improved morphology of the foot in relation to body weight with increasing age.

**Conclusions:**

This study shows that standardisation of the measurement protocol is imperative because results in typically developing children can vary depending up parameter selection and normalisation technique.

**Level of evidence:**

3

## Introduction

Given that an average adult takes ~6500 steps/day,^1^ over two million steps are taken by year. Moreover, the impact of foot strike and push-off results in forces that exceed the body weight by up to 50%.^2^ Consequently, the structures of the foot are subjected to high and repetitive stress. In a longitudinal study by Hill et al.,^3^ 20% of the participants experienced pain in the foot area. Deviations from the norm in the foot arch, in particular, have been identified as a significant risk factor for the development of pain and injuries to the lower extremities, including stress fractures and knee pain.^4^ Therefore, it is essential to examine foot deformities in childhood for potential impairment, while bearing in mind that diagnostics must account for the ongoing development in children.^5^

For instance, the longitudinal arch of the foot typically forms by the age of 10, meaning that flat foot is part of a normal development.^6^ As the fat pad under the midfoot recedes, bone ossification occurs, and motor control improves, the plantar pressure distribution changes.^7,8^ In general, plantar pressure can be measured using pedobarography. Due to its simple, quick and contactless procedure, good compliance can be achieved in children.^5^ In contrast with structural examinations using radiological imaging, pedobarography is also capable of capturing functional components, which differ from static properties due to additional muscle forces during gait.^9^ Furthermore, dynamic foot pressure measurement exhibits excellent test–retest reliability in both healthy and pathological adults and children.^10–13^ Therefore, pedobarography is an appropriate method for comparisons, which help to identify pathologies, conduct follow-up evaluations or determine the success of surgical interventions.^5^ Given the physical development that takes place from birth to adolescence, it is essential to utilise age-specific reference data to account for both structural and functional changes. In particular, the growth-related changes like increasing body weight can mask more subtle functional changes, when comparing different ages or the same individual over time. Normalised parameters are therefore superior to absolute values.^14^ Current literature describes techniques such as body weight normalisation^15^ or scaling with the maximum occurring value.^16^ However, it is unclear whether different scales could result in different conclusions, as no study used more than one normalisation. This makes comparisons between studies difficult, especially as there are few studies that cover the entire development from walking onset to adolescence. Therefore, conclusions about changes in plantar pressure can mostly be drawn from absolute values: both the maximum load (force and pressure) and, notably, the load on the forefoot increase.^15,17,18^ Additionally, the contact area increases in line with foot growth, while the contact area of the midfoot decreases relative to the total area.^7,15^ The loading patterns (foot region where peak pressure (PP) occurs) seem to develop beyond the age of 10 years.^15^ By the age of 15–17 years, foot development appears to be complete, as the mediolateral pressure distribution and force–time integrals (FTI) are similar to those of adults.^16^ Demirbüken et al.^19^ suggest that alteration in pressure patterns during early adolescence could be a risk factor for foot impairments.

Therefore, the aim of this cross-sectional study is to provide normative plantar pressure data for typically developing children aged 4–17 years to enable age-specific assessment of altered plantar pressure distributions. Additionally, different normalisations of the parameters will be evaluated to compare their explanatory power and ascertain whether disparate parameters measuring the total or peak load can be compared without compromising the conclusions drawn.

## Methods

This study was conducted in the gait laboratory of the Olga Hospital in Stuttgart, Germany. Children free of neuro-orthopaedic disorders aged 4–17 years were recruited by word of mouth as well as via a flyer on the hospital’s intranet. A total of 104 typically developing European children (48 girls, 56 boys aged 10.1 ± 3.6 years) participated in the study. The experimental protocol was approved by the Olga Hospital. The data were collected in compliance with the ethical principles of the Declaration of Helsinki (General Assembly of the World Medical Association, 2014). Detailed information about the measurement procedure was given and signed consent was obtained from the accompanying parents of the children with regards to collection, analysis and publication for this non-interventional study. Participants were allowed to leave the study at any point of time.

First, the parent or guardian of the child was requested to fill out a short questionnaire regarding medical history. The presence of lower extremity pain, underlying neurologic diseases or pathological foot conditions were exclusion criteria. Then, anthropometric data (age, gender, height, weight) were collected and feet photographed from all sides. A clinical examination was conducted by an experienced physiotherapist. Joint mobility, spasticity and muscle strength were evaluated,^20^ and an inspection of the feet was conducted to rule out the presence of foot deformities. Dynamic foot pressure measurement was performed using a 1440 × 440 mm^2^ pedobarography platform (emed^©^ xl, 4 sensors/cm^2^; Novel GmbH, Munich, Germany) with a measuring range of 10–1720 kPa. Two cameras (Logitech HD Pro Webcam c920, Lausanne, Switzerland) recorded the measurement area in the frontal and sagittal planes. Pressure and video data were collected using the emed^©^ Recorder software at 100 Hz (Novel GmbH, Munich, Germany, v28.3.34).

Using the midgait protocol,^21^ the subjects were asked to walk across the 6 m long walkway at their preferred walking speed. Trials were repeated until six usable footprints per foot (maximum one left and one right foot per trial) were obtained. A usable footprint is one where the foot hits the pressure plate cleanly and without abnormal events like tripping or intentionally trying to hit the platform.

In three participants, deviations in the walking pattern were identified through either visual observation or measuring outcomes. After discussion with the orthopaedist responsible for the gait laboratory, these cases were excluded from the study.

**Figure 1.**
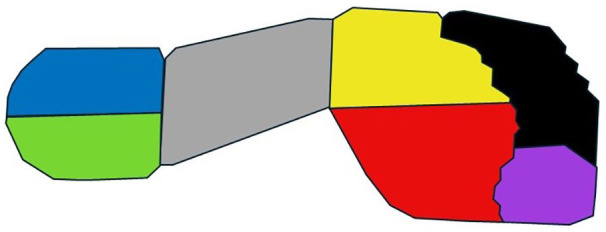
Subdivision of the foot with seven masks: lateral heel (blue), medial heel (green), midfoot (grey), lateral forefoot (yellow), medial forefoot (red), toes 2–5 (black) and hallux (purple).

The Novel scientific software package (Novel GmbH, Munich, Germany, v28) was used for further analysis. First, the foot was subdivided into seven regions or masks using the Novel Automask software: medial and lateral heel, midfoot, medial and lateral forefoot, hallux and toes (D2–5; Figure 1). The division of the heel and forefoot was based on the bisection of the plantar angle, which is defined by the tangents to the medial and lateral sides of the forefoot. Separation between the heel and midfoot is defined at 73% of the length from the toes to the heel, while the boundary between the forefoot and midfoot is set at 43%. To define the boundaries between the forefoot and the hallux or D2–5, the PP and its gradients in the surrounding regions were considered.

For each mask, the following parameters were subsequently calculated to analyse the peak load of the foot:

1. PP (kPa): PP (maximum pressure) occurring during the stance phase.
2. PP_BW_ (kPa/kg): PP normalised to body weight.
3. %PP (%): PP divided by the maximum pressure of the whole foot, also known as scaled PP.
4. *F*_max,BW_ (%BW): maximum force (*F*_max_) exerted during the stance phase, normalised by body weight.

To facilitate more effective interpretation of the parameters in terms of spatial shifts in the load over ageing, percentage of subjects experiencing PP in specific foot regions was analysed.

To analyse the total load during the stance phase, the following parameters were calculated:

5. FTI (N·s): area under the force-time curve.
6. FTI_BW_ (%BW·s): FTI normalised by body weight.
7. PTI (kPa·s): pressure–time integral.
8. PTI_BW_ (kPa·s/kg): PTI normalised by body weight.
9. PTI and PTI_BW_ were calculated from the FTI and FTI_BW_, divided by the area of the respective mask.^22^
10. PPTI (kPa·s): peak-PTI is defined as the sum of PPs overall.

Further data analysis was conducted using MATLAB (The MathWorks Inc., Natick, Massachusetts, R2023a). For this, the results of the six trials per foot were averaged, and either the left or right foot was randomly selected (*n*_left_ = 49, *n*_right_ = 52). This approach is necessary as pooling the data from both feet would violate the assumption of data independence in statistical analysis.^24^ Prior to this, a comparison between the left and right feet was performed using a t-test, which revealed no significant differences (*p* > 0.05) for any of the parameters. Then the children were divided into five age groups on the basis of developmental milestones and the results of prior studies^15,16^ with the groups being matched in size: 4–6, 7–8, 9–11, 12–14 and 15–17 years. After verifying normal distribution with the Kolmogorov–Smirnov test, a one-way ANOVA was conducted, followed by a post-hoc test with Bonferroni correction (α = 0.05) for significant results (*p* < 0.05) to compare the age groups.

## Results

Anthropometric data of the children are summarised in Table 1. The mean and standard deviation for each parameter and age group can be found in Tables 2 and 3.

**Table 1.** Anthropometric data of children (mean ± standard deviation).

Anthropometricmeasure	Age group				
	4–6	7–8	9–11	12–14	15–17
Weight (kg)	21.5 ± 3.1	26.4 ± 4.3	35.9 ± 6.8	52.4 ± 8.3	61.7 ± 12.7
Height (cm)	117 ± 7	130 ± 6	144 ± 7	164 ± 7	171 ± 7
Age (years)	5.4 ± 0.8	7.3 ± 0.5	9.7 ± 0.6	13.2 ± 0.8	15.7 ± 0.7
BMI	15.7 ± 1.6	15.5 ± 1.6	17.1 ± 2.0	19.4 ± 2.5	21.1 ± 3.5
Boys (%)	53	52	77	43	31
n	19	23	22	21	16

**Table 2.** Mean and standard deviation of *F*_max,BW_, PP, PP_BW_ and PP_scaled_ for each age group and foot region with *p* values of the ANOVA.

Parameter per foot region	Age group					*p* value
	4–6	7–8	9–11	12–14	15–17	
*F*_max,BW_ (%BW)						
Hallux	25.4 ± 8.3	22.2 ± 7.6	23.0 ± 9.0	24.3 ± 6.9	23.9 ± 11.0	0.793
Toes 2–5	10.5 ± 5.5	10.2 ± 6.8	8.1 ± 4.2	10.0 ± 3.9	10.1 ± 5.9	0.606
Medial forefoot	44.7 ± 6.3	47.6 ± 6.8	52.3 ± 8.5^a^	48.5 ± 8.2	51.7 ± 7.5	**0.014**
Lateral forefoot	45.2 ± 7.5	44.7 ± 10.2	45.5 ± 6.8	42.9 ± 6.9	44.0 ± 13.4	0.895
Midfoot	18.4 ± 8.3	18.4 ± 8.7	18.1 ± 10.0	17.2 ± 7.9	17.8 ± 9.9	0.992
Medial heel	46.0 ± 5.1	47.3 ± 6.3	47.1 ± 6.4	43.7 ± 6.2	44.1 ± 9.4	0.288
Lateral heel	41.0 ± 6.0	42.8 ± 6.4	41.1 ± 4.4	40.1 ± 4.7	40.1 ± 7.0	0.553
PP (kPa)						
Hallux	206.0 ± 103.9	218.6 ± 93.9	285.2 ± 124.4	346.6 ± 119.2^a,b^	365.3 ± 222.9^a,b^	**<0.000**
Toes 2–5	71.9 ± 27.9	92.8 ± 44.3	94.8 ± 58.9	105.5 ± 41.6	125.8 ± 71.4^a^	**0.034**
Medial forefoot	178.0 ± 51.8	218.7 ± 67.5	281.1 ± 104.3^a^	321.7 ± 106.4^a,b^	353.9 ± 102.7^a,b^	**<0.000**
Lateral forefoot	173.4 ± 53.4	229.0 ± 117.3	244.4 ± 93.8	282.8 ± 102.3^a^	299.5 ± 134.0^a^	**0.003**
Midfoot	90.7 ± 23.4	99.8 ± 27.9	92.1 ± 26.9	103.1 ± 32.2	113.2 ± 53.2	0.254
Medial heel	283.9 ± 49.3	357.5 ± 90.0	381.6 ± 109.3^a^	368.1 ± 112.9^a^	360.3 ± 67.9	**0.011**
Lateral heel	258.2 ± 44.3	328.7 ± 87.7	343.4 ± 90.8^a^	341.4 ± 101.0^a^	331.1 ± 61.7	**0.008**
PP_BW_ (kPa/kg)						
Hallux	97.8 ± 43.3	86.8 ± 41.1	83.0 ± 39.5	71.3 ± 32.7	62.2 ± 39.3	0.070
Toes 2–5	34.4 ± 13.3	37.3 ± 20.5	27.9 ± 16.9	21.5 ± 11.0^b^	21.9 ± 13.5^b^	**0.003**
Medial forefoot	84.0 ± 18.4	86.7 ± 30.5	82.5 ± 33.7	62.7 ± 17.4	60.6 ± 22.8^b^	**0.002**
Lateral forefoot	82.9 ± 26.5	91.0 ± 48.3	71.5 ± 30.3	54.3 ± 15.4^b^	52.0 ± 28.6^b^	**<0.000**
Midfoot	44.1 ± 13.8	39.5 ± 12.6	27.3 ± 10.1^a,b^	20.1 ± 5.7^a,b^	18.9 ± 9.0^a,b^	**<0.000**
Medial heel	137.1 ± 33.0	142.8 ± 45.6	112.3 ± 40.1^c^	73.5 ± 26.4^a,b^	61.8 ± 16.0^a [Table-fn table-fn3-18632521251335875]–c^	**<0.000**
Lateral heel	124.8 ± 30.6	131.7 ± 44.2	100.7 ± 32.8^c^	68.1 ± 23.7^a [Table-fn table-fn3-18632521251335875]–c^	56.5 ± 13.3^a [Table-fn table-fn3-18632521251335875]–c^	**<0.000**
PP_scaled_ (%)						
Hallux	0.6 ± 0.2	0.6 ± 0.2	0.7 ± 0.3	0.7 ± 0.2	0.7 ± 0.3	0.277
Toes 2–5	0.2 ± 0.1	0.2 ± 0.1	0.2 ± 0.1	0.2 ± 0.1	0.2 ± 0.1	0.974
Medial forefoot	0.6 ± 0.1	0.6 ± 0.2	0.6 ± 0.2	0.7 ± 0.2	0.7 ± 0.2	0.0491
Lateral forefoot	0.6 ± 0.1	0.6 ± 0.2	0.6 ± 0.2	0.6 ± 0.2	0.6 ± 0.2	0.924
Midfoot	0.3 ± 0.1	0.3 ± 0.1	0.2 ± 0.1	0.2 ± 0.1	0.2 ± 0.1	0.083
Medial heel	0.9 ± 0.1	0.9 ± 0.1	0.9 ± 0.1	0.8 ± 0.2^a,b^	0.7 ± 0.2^a [Table-fn table-fn3-18632521251335875]–c^	**<0.000**
Lateral heel	0.8 ± 0.1	0.9 ± 0.1	0.8 ± 0.1	0.7 ± 0.2^c^	0.7 ± 0.2^a,b^	**<0.000**

*F*_max,BW_: maximum force normalised to body weight; PP: peak pressure; PP_BW_: normalised peak pressure; PP_scaled_: scaled peak pressure.

a Significant difference to groups 4–6.

b Significant difference to groups 7 and 8.

c Significant difference to groups 9–11.

Significant results of the ANOVA are shown in bold.

**Table 3.** Mean and standard deviation of FTI_BW_, PPTI, PTI_BW_ and PTI for each age group and foot region with *p* values of the ANOVA.

Parameter perfoot region	Age group					*p* Value
	4–6	7–8	9–11	12–14	15–17	
FTI_BW_ (%KG·s)						
Hallux	4.5 ± 1.7	3.6 ± 1.3	4.3 ± 1.7	5.2 ± 1.8	5.4 ± 3.0^a^	**0.023**
Toes 2–5	1.7 ± 1.1	1.5 ± 1.3	1.2 ± 0.7	1.8 ± 0.8	1.9 ± 1.3	0.196
Medial forefoot	12.3 ± 3.7	12.1 ± 2.5	14.4 ± 2.5	14.3 ± 3.6	14.7 ± 2.50	**0.012** ^b^
Lateral forefoot	12.7 ± 1.8	12.8 ± 4.3	13.5 ± 2.6	13.8 ± 2.2	14.1 ± 4.8	0.636
Midfoot	3.3 ± 1.2	3.6 ± 2.3	4.0 ± 2.9	3.7 ± 2.3	4.1 ± 2.6	0.800
Medial heel	8.0 ± 1.8	8.5 ± 1.9	8.6 ± 1.4	8.5 ± 1.3	9.3 ± 2.0	0.242
Lateral heel	7.3 ± 1.8	7.6 ± 2.0	7.7 ± 1.2	7.9 ± 1.2	8.4 ± 1.7	0.337
PTI (kPa·s)						
Hallux	10.1 ± 2.8	10.1 ± 3.8	14.5 ± 6.1	21.0 ± 4.7^a,c,d^	25.3 ± 9.4^a,c,d^	**<0.000**
D2–5	4.0 ± 1.7	4.3 ± 2.6	4.1 ± 1.6	7.0 ± 2.0^a,c,d^	8.5 ± 3.8^a,c,d^	**<0.000**
Medial forefoot	15.1 ± 3.8	16.8 ± 3.3	23.9 ± 6.0^a,c^	32.0 ± 6.9^a,c,d^	38.0 ± 9.0^a,c,d,e^	**<0.000**
Lateral forefoot	14.2 ± 2.5	16.1 ± 4.3	21.0 ± 6.1	29.5 ± 7.6^a,c,d^	34.2 ± 14.2^a,c,d^	**<0.000**
Midfoot	4.3 ± 1.1	5.4 ± 2.5	6.5 ± 2.4	8.7 ± 3.3^a,c,d^	11.1 ± 5.7^a,c,d^	**<0.000**
Medial heel	15.2 ± 4.8	17.8 ± 6.1	21.3 ± 5.3	28.3 ± 5.7^a,c,d^	36.5 ± 0 11.3^a,c,d,e^	**<0.000**
Lateral heel	13.1 ± 4.3	15.1 ± 4.5	18.4 ± 4.5^c^	24.9 ± 4.8^a,c,d^	31.8 ± 9.9^a,c,d,e^	**<0.000**
PTI_BW_ (kPa·s/kg)						
Hallux	0.5 ± 0.2	0.4 ± 0.1	0.4 ± 0.2	0.4 ± 0.1	0.4 ± 0.2	0.270
D2–5	0.2 ± 0.1	0.2 ± 0.1	0.1 ± 0.0^c^	0.1 ± 0.0	0.1 ± 0.1	0.031
Medial forefoot	0.7 ± 0.2	0.7 ± 0.1	0.7 ± 0.1	0.6 ± 0.1	0.6 ± 0.1	0.178
Lateral forefoot	0.7 ± 0.1	0.6 ± 0.2	0.6 ± 0.1	0.6 ± 0.1	0.6 ± 0.2	0.119
Midfoot	0.2 ± 0.1	0.2 ± 0.1	0.2 ± 0.1	0.2 ± 0.0	0.2 ± 0.1	0.245
Medial heel	0.7 ± 0.2	0.7 ± 0.2	0.6 ± 0.1	0.6 ± 0.1^c^	0.6 ± 0.2	**0.017**
Lateral heel	0.6 ± 0.2	0.6 ± 0.2	0.5 ± 0.1	0.5 ± 0.1^c^	0.5 ± 0.2	**0.029**
PPTI (kPa·s)						
Hallux	101.3 ± 27.8	100.6 ± 38.2	145.5 ± 61.2	210.2 ± 47.1^a,c^	253.2 ± 94.5^a,c,d^	**<0.000**
D2–5	39.5 ± 16.9	43.2 ± 25.9	40.9 ± 15.8	70.4 ± 19.8	85.3 ± 38.3^a,c,d^	**0.001**
Medial forefoot	150.8 ± 37.9	167.8 ± 32.6	239.2 ± 60.3^c^	319.5 ± 68.8^a,c^	379.8 ± 90.2^a,c,d^	**<0.000**
Lateral forefoot	141.8 ± 24.6	161.0 ± 43.4	209.7 ± 60.9	295.0 ± 76.0^c^	342.4 ± 142.2^c^	**0.002**
Midfoot	43.4 ± 11.2	54.3 ± 24.5	65.1 ± 23.9	86.8 ± 32.8	110.5 ± 56.9	0.055
Lateral heel	130.8 ± 43.4	151.5 ± 45.1	183.5 ± 44.6^c^	248.9 ± 47.7^c^	318.0 ± 99.4^c^	**<0.000**
Medial heel	152.3 ± 47.9	177.5 ± 61.0	212.8 ± 52.7	283.2 ± 57.4^c^	365.1 ± 112.5^c^	**<0.000**

FTI_BW_: normalised force–time integrals; PPTI: peak pressure time-integrals; PTI: pressure–time integrals; PTI_BW_: normalised pressure–time integrals.

a Significant difference to groups 7 and 8.

b No significant differences in post-hoc comparison.

c Significant difference to groups 4–6.

d Significant difference to groups 9–11.

e Significant difference to groups 12–14.

Significant results of the ANOVA are shown in bold.

**Figure 2.**
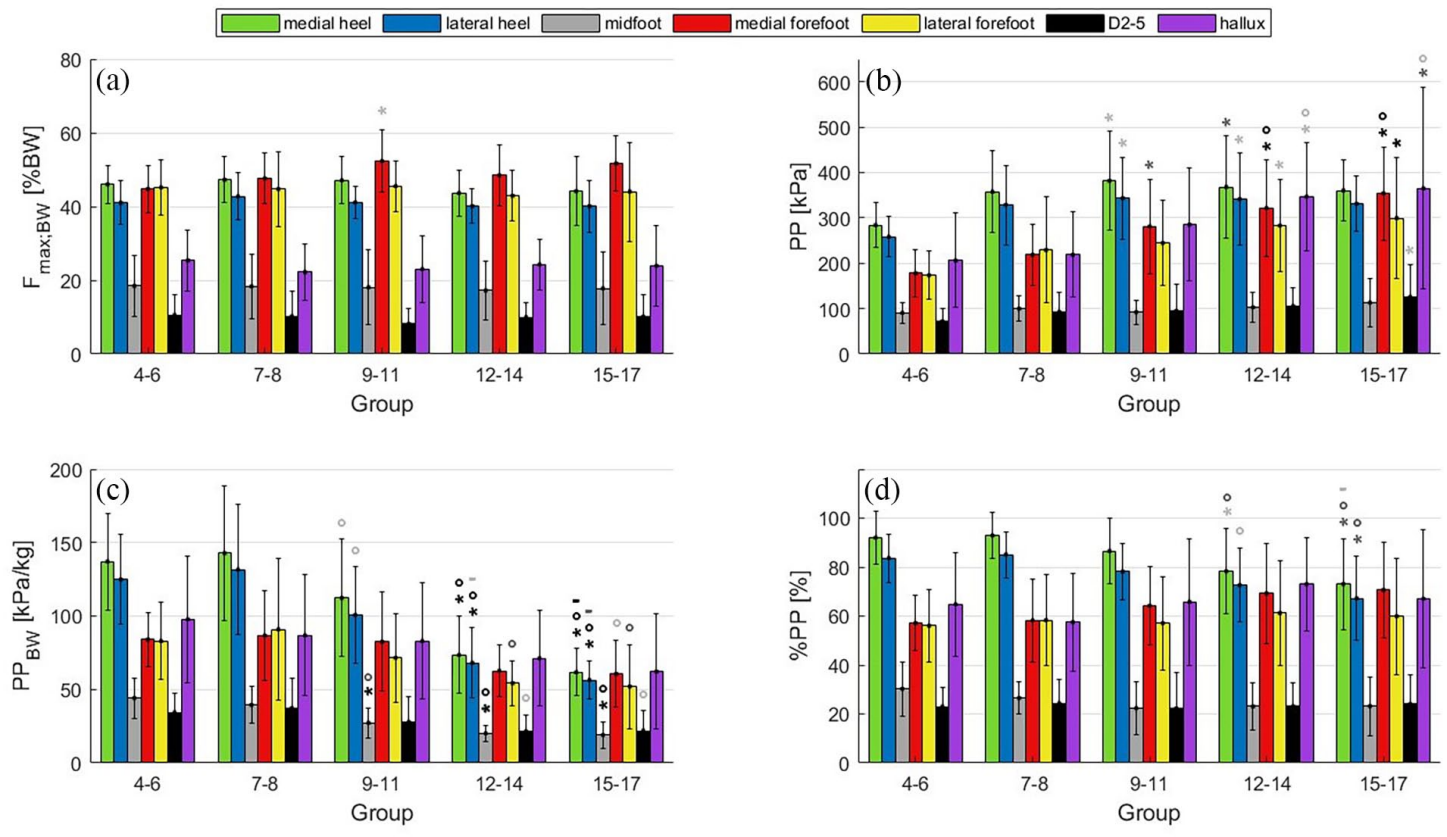
Peak load of the foot by age group and foot region: (a) *F*_max,BW_ (%BW), (b) PP (kPa), (c) PP_BW_ (kPa/kg), (d) %PP with ANOVA results. *F*_max,BW_: maximum force normalised to body weight; PP: peak pressure; PP_BW_: peak pressure normalised to body weight; %PP: peak pressure normalised to the maximum peak pressure. *Significant difference compared to groups 4–6. °Significant difference compared to groups 7 and 8. -Significant difference compared to groups 9–11. Light grey *p* < 0.05. Dark grey *p* < 0.01. Black *p* < 0.001.

*F*_max,BW_ remains nearly constant across the age groups with the highest forces occurring under the heel and forefoot (Figure 2(a)). In general, PP increases with age, except for the middle foot, which remains nearly constant around 100 kPa (Figure 2(b), grey bar and Table 2). There are almost no changes in PP between the younger two age groups (4–6 and 7–8 years) as indicated by the missing symbols for significant differences in Figure 2(b). When normalised for body weight, PP_BW_ is likewise similar for the two younger age groups (Figure 2(c)). Then there is a significant reduction in all PP_BW_ values with increasing age except for the hallux (purple bars). For example, PP_BW_ of the medial heel (Figure 2(c), light green bar) decreases by 50% from 145 kPa/kg at 7–8 years to 60 kPa/kg at 15–17 years. The scaled PP (%PP; Figure 2(d) and Table 2) shows a slight decrease from 85% to 67% and 92% to 73% only in the lateral (blue bars) and medial heel (green bars) of the older (12–14 and 15–17 years) age groups compared to younger age groups (4–6 and 7–8 years). The specific significance levels for each mask can be found in Figure 2.

**Figure 3.**
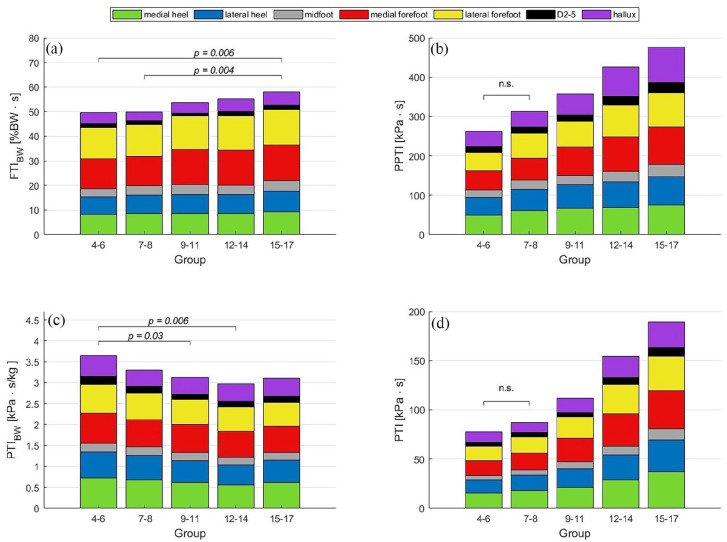
Total load during stance by age group and foot region: (a) FTI normalised to body weight (%BW·s), (b) PPTI (kPa·s), (c) PTI_BW_ (kPa·s/kg), (d) PTI (kPa·s). Significances are based on the value of the entire foot in (b) and (d) only the groups that do not differ significantly (n.s.) are indicated. FTI: force–time integral; PPTI: peak pressure–time integral; PTI: pressure–time integral; PTI_BW_: pressure–time integral normalised to body weight.

The parameters describing the total load during the stance phase (Figure 3), calculated as an integral, show similar trends to the peak load (Figure 2). However, when normalised to body weight, a significant increase from 49.8%BW·s to 57.9%BW·s in the normalised FTI (FTI_BW_) is observed in the 15–17 years (Figure 3(a)) compared to the two younger age groups (4–6 and 7–8 years). Similar to the results for PP (Figure 2(b)), PTI (Figure 3(d)) and PPTI (Figure 3(b)) show significant increases but no changes for the two youngest age groups. Note that only non-significant differences are marked in Figure 3(b) and (c), for better clarity. In contrast, the PTI_BW_ (Figure 3(c)) is similar to the PP_BW_ and is significantly higher in the 4–6 years than in the 9–11 and 12–14 years age groups and drops from 3.6%BW·s to 3.0%BW·s.

**Figure 4.**
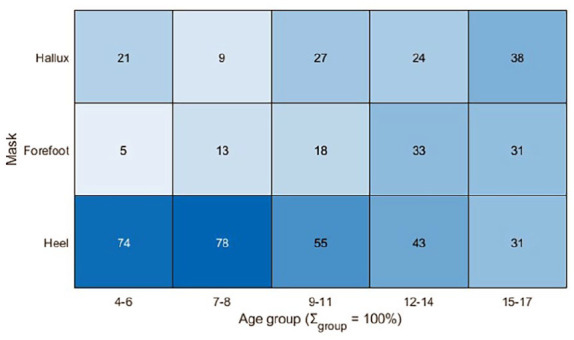
Percentage of subjects experiencing peak pressure in specific regions/masks (heel, forefoot, mask), note that the sum per age group is 100%.

Figure 4 shows that the area where PP occurs becomes more variable with increasing age. In children aged 4–8 years, three quarters have PP under the heel. In the 9–11 age group, this drops to half, and at 15–17 years of age, PP occurs almost equally under the forefoot, hallux and heel.

## Discussion

With this study, we were able to provide a set of plantar pressure data using several normalisation methods for the first time. In general, almost all parameters changed with age. The non-standardised values tended to increase, for example, PP and PTIs. The standardised values tended to decrease, when normalised to bodyweight, with the exception of FTI_BW_, which increased. Exceptions were the parameters *F*_max,BW_, which remained more or less unchanged and %PP, which showed no clear trend across foot regions.

### Comparison with the literature

We found that PP increases with age. This is similar to Müller et al.,^18^ who observed an increase of PP under all foot regions in a cross-sectional study with 10,382 healthy German children (1–13 years). Bosch et al.^15^ were also able to show this trend in a longitudinal study design that followed 36 healthy children from walking onset for 10 years. When normalised to bodyweight Unger and Rosenbaum^14^ found increasing PP_BW_. This contradicts our results, but they only looked at infants. In particular, major changes in the foot arch are observed until the age of 6.^15^ The pooling of the age groups from 4 to 6 may explain why the averaged PP did not reveal any differences in the midfoot load between the youngest group and the older children (Table 2). In contrast to nearly constant *F*_max,BW_ values across the age groups observed in our study (Figure 2(a)), Bosch et al.^15^ found increasing values in the hindfoot, forefoot, hallux and D2–5. However, they also found a load shift to the forefoot which is consistent with our findings and those of McKay et al.^25^

In terms of total load, our findings indicate that PTI and FTI increase with age. This is in line with McKay et al.,^25^ who found a moderate to strong positive correlation between age and PTI and FTI of the fore- and hindfoot and Kasović et al.^26^ who found increasing PTIs in 1284 children aged 6–14 years. The use of PTI_BW_ has not been reported in the existing literature.

In total, we observed the most pronounced changes between the age of 7 and 14. This is in accordance with Dulai et al.,^16^ who found no significant differences in medio–lateral FTI ratios and regional FTI between 15 and 17 year olds and adults. This suggests that functional and skeletal development is complete by the age of 15–17 years.

### Impact of functional changes on PP

Unadjusted peak loads and integrals show a strong increase in foot loading with increasing age. This is already known and is mainly attributed to increasing body weight.^25–27^ Results from different normalisations suggest additional effects due to physical maturation. The increase in additional forces to body weight represented by FTI_BW_ indicates a more dynamic gait pattern,^15^ supported by the shift of peak forces to the forefoot region.^15,17,18^ Morag and Cavanagh^28^ identified functional characteristics in 29 adults that reduce the load under the heel, including prolonged ground contact time, which increases with age^26^ and reduced approach velocity at initial contact. Liu et al.^29^ suggest that the approach velocity may be higher in children due to less control over deceleration compared to adults. These functional changes could compensate for the increase in weight and explain why PP of the heel only increases up to the age of 9–11 years. As these predictors of PP were identified in adults, this explanatory approach cannot be fully confirmed. Furthermore, the significant decrease in %PP under the heel underpins that the relative load in the forefoot increases due to a more dynamic gait pattern.^15^ Male subjects achieved significantly higher plantar flexion moments at the ankle from the age of 8, allowing for a stronger push-off from the ground in the terminal stance phase.^30^ McKay et al.^25^ also showed in a study with 16 adolescents aged 10–19 years a positive correlation (*r* = 0.4) between the strength of the plantar flexors and the PP in the forefoot, while the strength did not affect the PP in the heels. The transfer of force during push-off from the ground might also improve through the stabilisation of the arch,^31^ resulting in overall higher PPs in the forefoot.

### Impact of body weight normalised pressure parameters

The body weight normalised pressure parameters surprisingly showed a decrease with increasing age. Unlike *F*_max,BW_ and FTI, pressure also takes into account the area over which the forces act. As the area of the foot increases more in relation to the normalised forces, the latter are distributed over a larger area. This effect diminishes from around 14 years of age, probably because weight, unlike foot size, continues to increase beyond adolescence.^26,32,33^ This explanation is supported by the changes in plantar forces in overweight individuals. Here, these proportions are altered, leading to increased loads on the feet,^34^ as the musculoskeletal system cannot fully compensate for the additional forces.^35^ In particular, the midfoot is subject to greater stress due to increased compression of the arch.^15,35,36^ In normal development, its load changes little with age, and relative to the total load, the midfoot load actually decreases as the arch develops.^7,16^ McKay et al.^25^ showed that in contrast to the forefoot and hindfoot, peak midfoot pressure does not correlate with age in children aged between 3 and 19 years. This could be because the midfoot is subjected to very few deceleration or acceleration forces other than the weight force. The same applies to D2–5, which are not involved in the more dynamic phases of walking (initial contact, terminal stance).^37^

### Importance of a standardised measurement protocol

Since foot loading (PP, PPTI, PTI) depends heavily on body weight, which in turn is related to age, appropriate normalisation is crucial for cross-age comparisons in children. However, the results are highly dependent on the normalisation method. When normalising with the maximum occurring value, effects of body weight can mix with relative effects of foot regions if the peak value itself depends on body weight. Thus, normalisation with body weight might be best suited for cross-age comparisons. In the calculation of the PPTI, although the area is considered, only the sensor information with the maximum load is used per frame. Thus, it is no longer possible to distinguish how large the area was where high forces acted. This leads to the PPTI values increasing less sharply than the PTI and underestimating the change in overall loading (see Figure 3(b) and (d)). Melai et al.^22^ compared PPTI and PTI values for patients with diabetes and concluded that PTI is better suited for assessing the risk of foot ulcers. There are no data yet for other foot pathologies, but it is likely that this problem also occurs in the assessment of pes planus. Due to the flattening of the foot arch, the midfoot experiences an increase in the contact area, while the PP shows no significant changes.^23^ The pressure changes across age observed in the lateral and medial regions of the heel and forefoot are similar. This could give the impression that the subdivision is redundant. However, in foot pathologies, the mediolateral loading is altered, justifying the more differentiated analyses.^38^ Overall, the corresponding parameters for peak and total load (e.g. PP_BW_ and PTI_BW_) behave similarly in healthy children, but it is unclear whether this is also the case for pathological footprints. In contrast, parameters that appear to describe the same load exhibit disparate behaviour when analysed using different normalisation techniques. For instance, the parameters PP, PP_BW_, %PP and *F*_max,BW_ appear to quantify the peak load, yet they display distinct trends. Consequently, when comparing measurements, it is essential to consider the specific parameter under evaluation to ensure accurate and meaningful comparisons.

### Limitations

The results of this study may be affected by certain limitations. Walking speed was not controlled, although it can affect plantar pressure.^13,39,40^ However, Müller et al.^18^ observed no variation in walking speed within age groups in the assessment of pedobarographic parameters. Furthermore, controlling cadence would alter natural gait patterns, reducing the validity of the results. Gender-specific differences were not analysed due to the small sample size. Although the normalisation of the parameters largely eliminated the differences in body weight development during puberty and post-adolescence, it did not account for the significant influence of puberty on musculoskeletal development. This might yield a large variability in the measured parameters and may influence statistical outcomes. Thus, future studies should include a larger sample size to examine smaller age groups, and, if possible, investigate gender-specific differences.

## Conclusion

To the best of our knowledge, this is the first study to use multiple normalisation techniques, covering the entire development from walking onset to adolescence. This allows for a comparison of changes in different normalisation techniques for measuring peak and total load and for an assessment of their explanatory power. The total plantar pressure increases with age, predominately due to increasing body weight and a more dynamic gait pattern. In contrast, normalisation to body weight yielded decreased PP and pressure time integrals suggesting an improved morphology of the foot in relation to body weight with increasing age. The normative values for quantitative measurement are already in clinical use at the gait laboratory of the Olga Hospital in Stuttgart, Germany for age-specific identification of pathologies, interpretation and postulation of primary and secondary causes and recommendation of treatment. However, the findings indicated that the comparison of parameters characterising peak or total load can become unreliable when different normalisation techniques are employed, complicating accurate and meaningful comparison of data from different gait laboratories. Given that different normalised parameters diverge even in healthy children, a standardised measurement protocol should be developed.

## Supplemental Material

sj-pdf-1-cho-10.1177_18632521251335875 – Supplemental material for Impact of body weight and age on plantar pressure in typically developing children: Normative data and methodological considerations Supplemental material, sj-pdf-1-cho-10.1177_18632521251335875 for Impact of body weight and age on plantar pressure in typically developing children: Normative data and methodological considerations by Anika Behrendt, Tobias Siebert and Sonia D’Souza in Journal of Children’s Orthopaedics
